# Terahertz antiferromagnetic dynamics induced by ultrafast spin currents

**DOI:** 10.1126/sciadv.adx1107

**Published:** 2025-11-07

**Authors:** Sanjay René, Artem Levchuk, Amr Abdelsamie, Zixin Li, Pauline Dufour, Arthur Chaudron, Florian Godel, Pascale Gemeiner, Brahim Dkhil, Jean-Baptiste Moussy, Karim Bouzehouane, Stéphane Fusil, Vincent Garcia, Michel Viret, Jean-Yves Chauleau

**Affiliations:** ^1^ SPEC, CEA-Saclay, CNRS, UMR3680, Université Paris-Saclay, 91191 Gif-sur-Yvette, France.; ^2^ Laboratoire Albert Fert, CNRS, Thales, Université Paris-Saclay, 91767 Palaiseau, France.; ^3^ Laboratoire Charles Coulomb, Université de Montpellier and CNRS, 34095 Montpellier, France.; ^4^ Laboratoire SPMS, CentraleSupélec, CNRS, Université Paris-Saclay, UMR8580, 91192 Gif-sur-Yvette, France.; ^5^ Université d’Evry, Université Paris-Saclay, 91000 Evry, France.

## Abstract

Insulating antiferromagnets are anticipated as the main protagonists of ultrafast spintronics, with their intrinsic terahertz dynamics and their ability to transport spin information over long distances. However, ultrafast transfer of spin angular momentum to an antiferromagnetic insulator remains to be demonstrated. Here, studying the picosecond and subpicosecond dynamics of ferromagnetic metal/antiferromagnetic insulator bilayers, we evidence the generation of coherent terahertz excitations in the antiferromagnet combined with a modulation of the demagnetization behavior in the ferromagnet. We thus demonstrate that magnetic information can indeed be propagated into antiferromagnetic spin waves at picosecond timescales, thereby opening an avenue toward ultrafast manipulation of magnetic information.

## Teaser

Terahertz spin information is transferred to antiferromagnetic insulators.

## INTRODUCTION

Antiferromagnets (AFs) (*1*), in which neighboring atomic magnetic moments are antiparallelly aligned, are now being considered as active components of the next generation of information and communication technologies. In the past decade, AFs have become key players in spintronics (*2*, *3*), due to efficient interactions between AF spin textures and spin currents, i.e., currents carrying spin angular momentum, cornerstone of spin information processing. A particular attention has been devoted to insulating AF materials, as they are expected to transport spin information at low-energy cost and high frequencies.

AFs exhibit superior dynamics, as their magnetic resonances (*4*, *5*) are intrinsically in the terahertz (*6*, *7*) range and the intrinsic damping of insulating compounds can be substantially low. This implies that their magnetic textures can be naturally actuated at picosecond timescales. Mastering and extending the spintronic concepts to subpicosecond timescales using AF terahertz dynamics would pave the way for tomorrow’s energy-efficient ultrafast devices.

A first AF spintronic fundamental concept is the transport of spin information through AF insulators (*8*–*12*). This has been demonstrated in both thin films and single crystals from DC to gigahertz, which is far from their natural subterahertz and terahertz resonance frequencies. These pioneering studies have shown that spin fluctuations in the AF can potentially propagate spin angular momentum as evanescent waves in AF insulators (*13*). The propagation may occur over long distances depending on the characteristics of the linear AF modes (*14*) and the size and quality of the samples involved. A second important spintronic notion, the so-called spin pumping (*15*) where spin currents can be dynamically generated by magnetic resonance, has been theoretically discussed in typical heavy metal/insulating AF bilayers (*16*). Pioneering experiments (*17*, *18*) reported AF spin pumping at subterahertz frequencies, emphasizing the ultrafast performance of AF spin textures. In the same vein, Rongione *et al.* (*19*) have shown that this can be extended to higher frequencies by measuring the terahertz emission of Pt/NiO bilayers, NiO being a prototypical insulating AF.

Nevertheless, direct evidence of spin angular momentum transfer or spin transfer torque (STT) to an insulating AF at picosecond and subpicosecond timescales is still missing. Because of vanishing net magnetization in AF textures, direct measurements of AF dynamics or AF magnons are extremely challenging, especially in thin films.

**Fig. 1.**
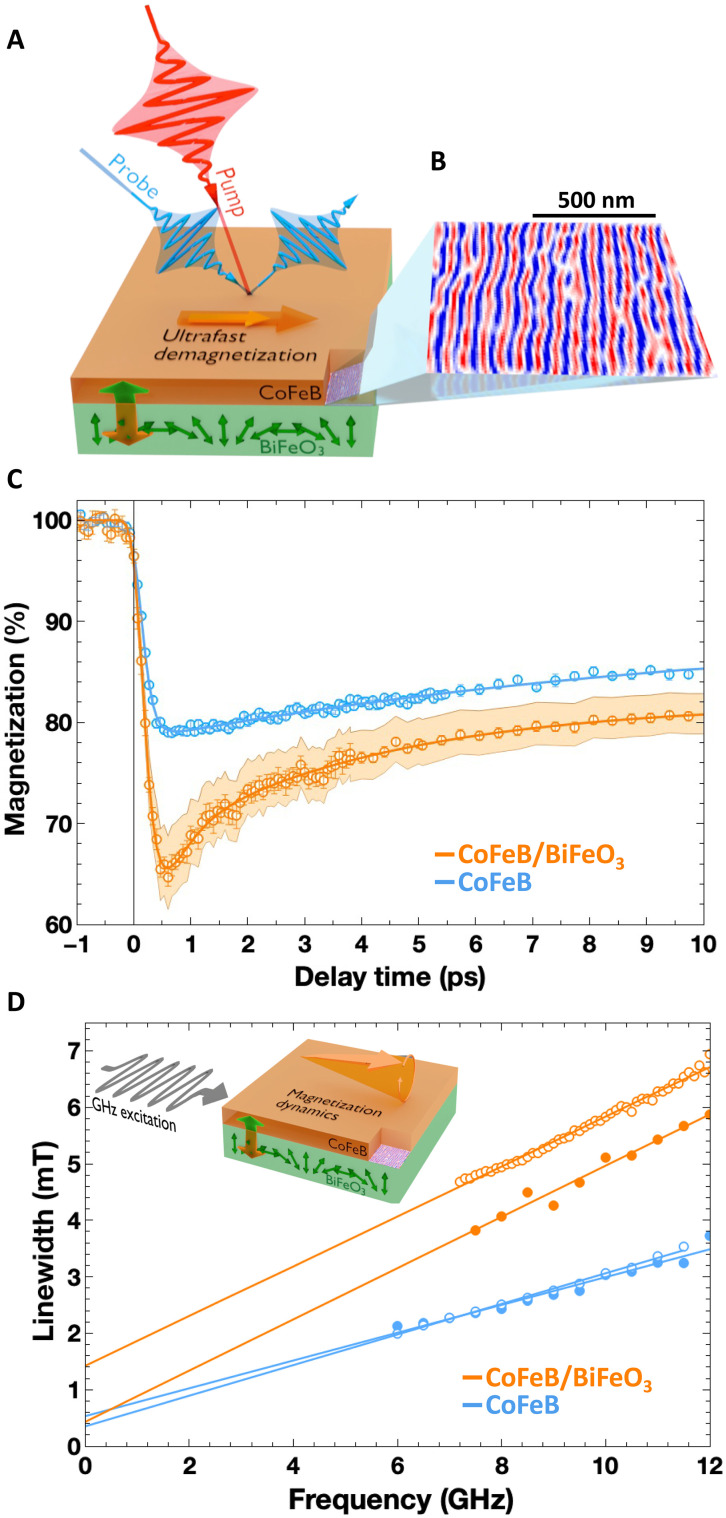
Additional spin angular momentum dissipation channels: from gigahertz to terahertz. (**A**) Schematic representation of the time-resolved magneto-optical Kerr effect (Tr-MOKE) experimental configuration. The large bicolor double arrow stands for the ultrafast exchanges of angular momentum between the ferromagnet and the AF. (**B**) Scanning nitrogen-vacancy (NV) magnetometry image of the single AF cycloidal state present in the epitaxial BiFeO_3_ layer with a typical period of 67 nm. (**C**) Ultrafast magnetization dynamics of the CoFeB/BiFeO_3_ bilayer (orange dots) and the CoFeB reference layer (blue dots). The CoFeB magnetization and the probe incidence plane are along the BiFeO_3_ cycloid plane or the DyScO_3_ *a* axis, and the probe is p polarized. In this configuration, the BiFeO_3_ is not probed. Therefore, the magnetization variation is presented as ΔMM0 in percent (%) (details about the calibration can be found in note S1). (**D**) Ferromagnetic resonance (FMR)/spin-pumping measurements performed on the CoFeB reference (blue dots) and the CoFeB/BiFeO_3_ sample (orange dots). For each sample, two configurations are considered with the external magnetic field applied either along (opened dots) or perpendicular (full dots) to the *a* axis of the DyScO_3_ substrate (also, parallel to the propagation direction the BiFeO_3_ cycloid).

Here, we tackle this issue by measuring the ultrafast dynamics of ferromagnetic/insulating AF bilayers excited by an intense femtosecond optical pulse and probed using ultrafast time-resolved magnetooptics. The strategy is to assess the effect of an exchange of spin angular momentum with the insulating AF texture on the ultrafast demagnetization characteristics of the ferromagnetic layer and detect the triggered terahertz dynamics in the AF layer (Fig. 1A).

Since its experimental evidence in 1996 by Beaurepaire *et al.* (*20*), the underlying mechanisms of laser-induced magnetization quench on subpicosecond timescales have been extensively studied (*21*–*24*). Although open questions still remain, various phenomena at play have been identified. These include scattering processes involving spin-flip events (*25*), transfer of angular momentum to other reservoirs such as phonons, optically induced spin transfer between different atomic sites or elements known as OISTR (*26*, *27*), and, lastly, spin transport (*28*–*31*), as ultrafast spin currents can circulate between the ferromagnetic and adjacent layers. Different kinds of ultrafast spin currents exist. On one hand, super diffusive hot carriers generate a spin current stemming from the different light excitations of majority and minority spins (*29*, *31*, *32*). On the other hand, exchange interactions between electrons separated into “s” (transport) and “d” (local magnetization) produce a subpicosecond analog to the ferromagnetic resonance (FMR) spin pumping (*33*, *34*). These spin currents can be used, for example, to induce spin dynamics into a second ferromagnetic layer (*35*) or to generate a transient charge current in a large spin-orbit coupling material, as spins are converted into charge, triggering a terahertz radiation (*36*–*38*). The ferromagnetic layer can be therefore considered as an ultrafast spin current generator. Any kind of exchange of spin angular momentum with an adjacent insulating AF layer is expected to modify the intrinsic ultrafast demagnetization characteristics of the ferromagnet. Here, we measure the influence of an adjacent insulating AF: BiFeO_3_ thin film on the demagnetization dynamics of an ultrathin metallic CoFeB and evidence the terahertz dynamics in the AF triggered by ultrafast STT.

## RESULTS

For the purpose of this work, an AF material with a well-established resonance in the terahertz range and a Néel temperature (*T*_N_) far above room temperature is required. Any substantial sensitivity to thermal effects would hinder the observation of an additional transfer of angular momentum. While prototypical NiO is a good candidate, with a 1-THz AF resonance and *T*_N_ = 523 K (in bulk crystals), controlling its AF microscopic domain configurations in thin epitaxial layers remains challenging. Instead, the magnetoelectric AF BiFeO_3_ (*39*) with an AF dynamics between 0.5 and 1 THz (*40*) and *T*_N_ = 650 K is the material of choice for our study. The most salient consequence of this magnetoelectric coupling is the stabilization of an AF spin texture in the form of a well-characterized spin cycloid (*39*). In thin films, the AF cycloidal state can be controlled by epitaxial strain engineering (*41*, *42*), and recently, BiFeO_3_ layers harboring a single AF cycloidal domain (along with a single ferroelectric domain) have been produced (Fig. 1B and Materials and Methods) (*43*). Because the AF spin texture is homogeneous throughout the whole sample, the complexity of averaging multiple AF domains is avoided, and angular variations can also bring important extra information. Besides, the polar nature of the material brings another advantage for the detection of the ultrafast dynamics of the AF spin texture. In BiFeO_3_, thanks to the magnetoelectric interaction, the AF dynamics is associated to a polar dynamical contribution, which is more easily measurable, thanks to the strong birefringent nature of BiFeO_3_. This allows us to experimentally demonstrate the coherent ultrafast AF response triggered by STT.

### Incoherent spin transfer to the AF spin texture: From gigahertz to terahertz

The system’s dynamics at the picosecond and subpicosecond timescales is assessed using a time-resolved magneto-optical Kerr effect (Tr-MOKE) pump-probe setup. Figure 1C shows the ultrafast change in magnetization as a function of time on both the reference CoFeB and CoFeB/BiFeO_3_ layers. First of all, while our time resolution does not allow for the observation of a substantial difference in the demagnetization rates of the two systems ( ≈200 fs), a significant enhancement of the demagnetization amplitude is observed in the CoFeB/BiFeO_3_ bilayer. For an equivalent absorbed pump energy density in the CoFeB layer, the demagnetization amplitude of about 20% in the single CoFeB reference layer is substantially increased to 35% in the CoFeB/BiFeO_3_ bilayer (Fig. 1C and note S2). This is a clear signature of the opening of an additional ultrafast dissipation channel due to the adjacent BiFeO_3_ layer. The second notable difference appears in the remagnetization processes. For the reference CoFeB layer, the remagnetization behavior is reminiscent of a unique spin-to-phonon dissipation channel with a characteristic time of 10 ps. In contrast, the remagnetization of the CoFeB/BiFeO_3_ bilayer can only be understood by considering two remagnetization processes, with characteristic times of about 0.5 and 6 ps (fig. S5). The latter has the same phononic origin as that of the single CoFeB layer albeit with a slightly shorter characteristic time denoting a stronger coupling to the lattice due to the addition of BiFeO_3_. The short timescale remagnetization contribution evidences the opening of an additional angular momentum dissipation channel that enhances the damping of the high-energy magnons generated during the demagnetization process. This is the signature of the ultrafast pumping of a spin current to the AF insulator. We ruled out a potential contribution of the ferroelectric order in the fast remagnetization process by showing (see fig. S10) that no such behavior is observed for a CoFeB grown on a pure ferroelectric crystal. This is not surprising as we can expect that coupling between two magnetic systems, moreover at their resonance frequencies, should be stronger than any magnon/phonon mechanism. In addition, the role of incoherent spin fluctuations in the AF layer can also be probed far from the terahertz AF resonances. This is done using spin pumping during FMR, which can measure the additional magnetic dissipation induced in the ferromagnet because of the proximity with the AF. The magnetic damping provides a clear signature of the propensity of the AF order to absorb the angular momentum generated during FMR (*44*). Figure 1D shows such measurements performed on both the CoFeB/BiFeO_3_ bilayer and the CoFeB reference layer comparing the FMR linewidth as a function of the excitation frequency ( f ) for the two samples (details in note S3). They both exhibit characteristic linear behaviors from which one can infer the magnetic damping coefficient ( α ) and the inhomogeneous contribution ( $\mu_{0}\Delta H_{0}$ ), following: μ0ΔH=2π(αγ)·f+μ0ΔH0 , where γ is the gyromagnetic ratio and $\mu_{0}$ the vacuum permeability. While the magnetic damping coefficient for the CoFeB reference layer is $\alpha_{\text{ref}}=7.5\times 10^{-3}$ , one of the CoFeB/BiFeO_3_ bilayer is $\alpha_{\text{BFO}}=13\times 10^{-3}$ , implying that an additional $\Delta \alpha =5.5\times 10^{-3}$ contribution to the damping stems from spin angular momentum dissipated via an additional channel mediated by AF fluctuations. This is consistent with what has been observed (*8*, *44*) in other ferromagnet/AF bilayers. Besides, we also find that the intrinsic magnetic damping is isotropic (Fig. 1D). This points to an AF spin fluctuation origin for the additional dissipation mechanism, rather than coherent excitations of AF dynamics (even far from their resonance frequencies). As the BiFeO_3_ layer hosts one cycloidal domain, noticeable angular variations could be expected in the coherent picture: When the injected angular momentum is perpendicular to the AF cycloidal plane, the STT is equivalent on all the magnetic moments, while, when the angular momentum is parallel to the cycloidal plane, the STT follows a sinusoidal dependence along the magnetic moments of the cycloid, leading to a lower Δα.

Consequently, the isotropy of the damping indicates that at the nanosecond timescale, incoherent spin fluctuations in the AF are the main cause for the angular momentum exchange. The observations in Fig. 1C result from similar mechanisms but on a different timescale. The ratio $\alpha_{\text{BFO}}/\alpha_{\text{ref}}$ is identical to the demagnetization amplitude ratio between the CoFeB/BiFeO_3_ and CoFeB, thus hinting at a similar nature for the effect yet extended to the ultrafast regime.

### Coherent terahertz spin excitation of the AF texture

At the picosecond, however, the situation can be quite different, as this timescale matches the resonant excitations of AF magnons. To evidence a possible coherent triggering, one needs to access the AF dynamics. In the case of magnetoelectric materials such as BiFeO_3_, a polar character is associated to the AF dynamics (see note S4), which enables its detection through dynamical changes of birefringence [see for example Khan *et al.* (*45*)]. A thorough study of different geometries (parallel/perpendicular to the cycloid, p- or s-polarized light, transverse or longitudinal MOKE; see note S4) allows disentangling contributions coming from either CoFeB or BiFeO_3_ dynamics.

**Fig. 2.**
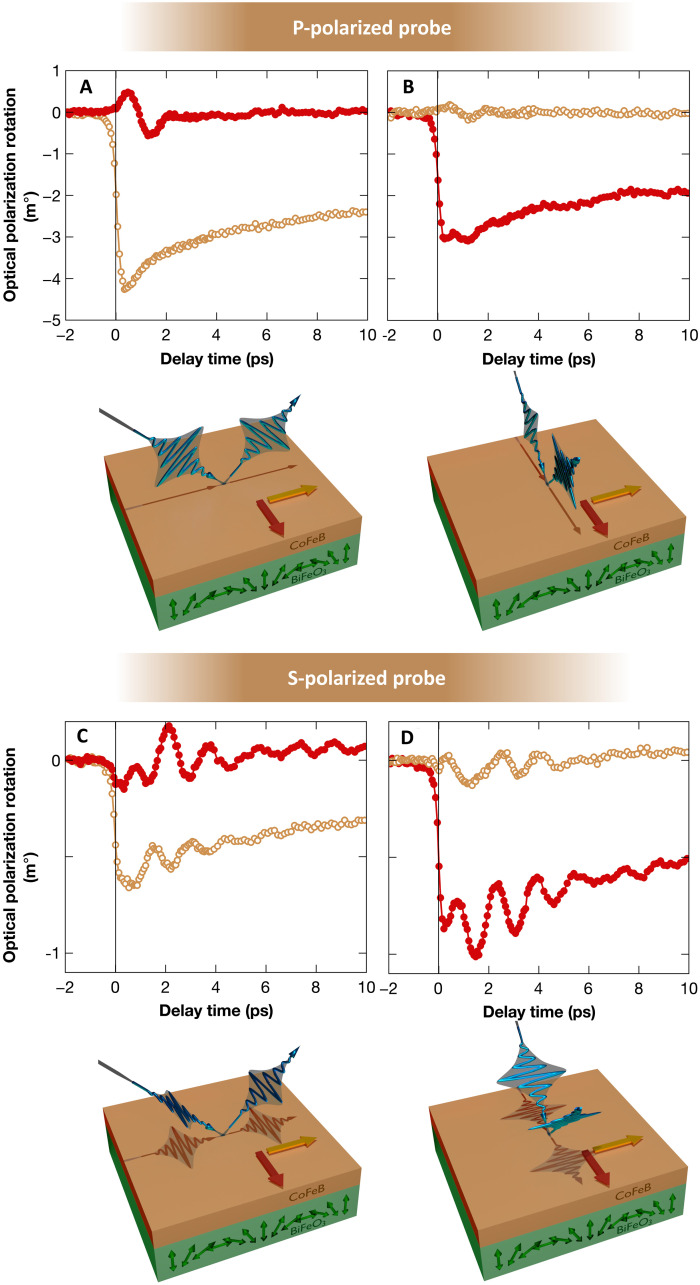
Comparison of the CoFeB/BiFeO_3_ bilayer ultrafast dynamics with respect to the probe polarizations. (**A** and **B**) P-polarized probe. (**C** and **D**) S-polarized probe. The plane of incidence of the probe beam and that of the BiFeO_3_ cycloidal are either coinciding (A and C) or orthogonal (B and D). For each panel, two color codes for the data define two experimental configurations with the CoFeB magnetization either parallel (orange) or perpendicular (dark red) to the propagation vector of the BiFeO_3_ cycloid, as defined in the corresponding sketches.

The four possible magneto-optical configurations are gathered in Fig. 2. All measurements evidence some terahertz dynamics. The s-polarized probe optimally shows the emergence of a signal at about 0.6 THz (Fig. 2, C and D), in agreement with typical electromagnon frequencies in BiFeO_3_ (*40*), which depends on the relative orientation between the injected spin current and the AF cycloidal plane. We recall here that to exclude any possible parasitic optical effects (fig. S8), the extracted signals are always the difference between opposite magnetization directions, which is a standard approach in Tr-MOKE studies. In the present case, this means that the phase of the observed subterahertz oscillations is odd with respect to the direction of the CoFeB magnetization, i.e., it follows the direction of the induced STT.

**Fig. 3.**
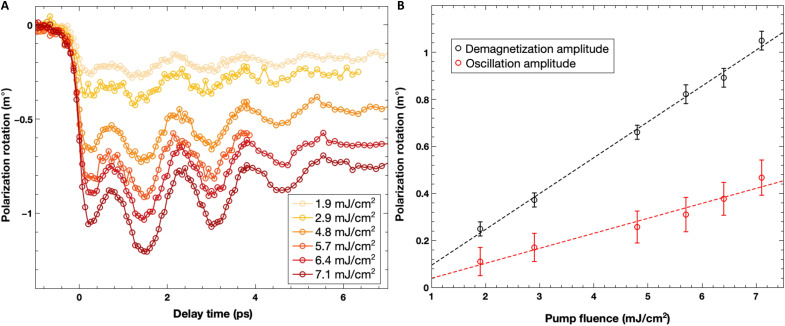
Dependence of the ultrafast dynamics on the incident pump fluence. (**A**) Ultrafast dynamics of the CoFeB/BiFeO_3_ measured for an s-polarized probe beam with the CoFeB magnetization set in the incident probe plane and perpendicular to the BiFeO_3_ AF cycloidal plane (equivalent to the geometry of Fig. 2D, red curve). (**B**) CoFeB demagnetization amplitude and BiFeO_3_ terahertz oscillation amplitude as function of the incident pump fluence.

These observations demonstrate the importance of the relative angle between the injected spin current and the AF cycloidal plane, emphasizing the ultrafast STT origin of the triggered AF dynamics. Exchange bias and anisotropy can enhance the transfer of spin angular momentum to the AF texture, as reported in the DC/gigahertz regime (*8*, *13*). AF dynamics is also triggered when the CoFeB magnetization is set along the easy axis. Because in this configuration, the quench of ferromagnetic magnetization does not apply any torque via a change of in-plane anisotropy related to the interfacial exchange, this confirms that STT is indeed the main source of AF dynamics. Moreover, direct optical pumping in the BiFeO_3_ layer is discarded by the observation that the pump polarization has no influence on the observed dynamics (fig. S9) and considering as well that most of the pump power is absorbed in the CoFeB layer. Last, the incident pump fluence dependence of the ultrafast dynamics (Fig. 3A) has been measured in the configuration where both the CoFeB demagnetization and the AF terahertz oscillation in BiFeO_3_ can be self-consistently assessed (i.e., s-polarized probe/CoFeB magnetization in the incidence probe plane and perpendicular to the AF cycloidal plane). Both demagnetization and oscillation amplitudes scale linearly with the incident pump fluence ruling out a potential direct excitation of the BiFeO_3_ layer via two-photon absorption processes (Fig. 3B).

### Simulation of the STT-induced terahertz dynamics of an AF cycloid

**Fig. 4.**
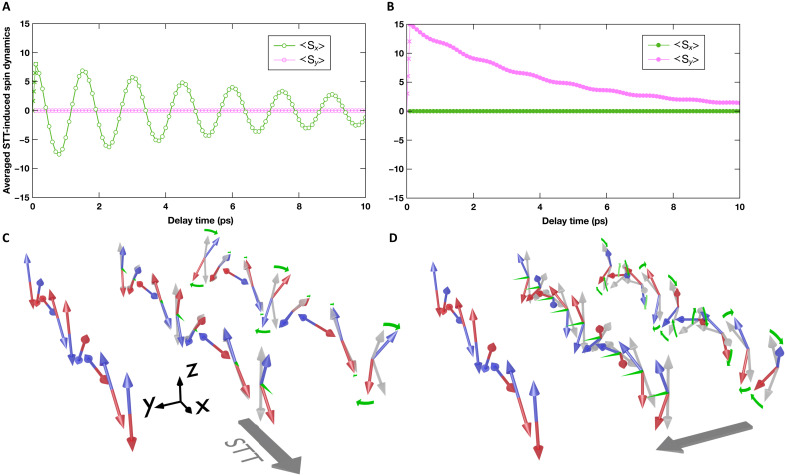
Atomic spin simulations of the STT-induced AF cycloid dynamics. (**A** and **B**) Averaged magnetic moment components when the STT is applied along (A) and perpendicular (B) to the AF cycloidal plane. (**C** and **D**) Sketches of their corresponding early-stage spin dynamics for the STT along or perpendicular to the cycloidal plane, respectively.

The experimental assessment of the exact nature of the STT-induced AF dynamics from the measured dynamical birefringence is challenging. Nonetheless, important additional insights can be obtained by performing atomic spin simulations using a Hamiltonian in which all relevant energy terms have been included [details can be found in (*46*)]. The pump energy is well below the BiFeO_3_ bandgap, and the pump fluence is only a few millijoules per square centimeter. Therefore, we consider that BiFeO_3_ keeps its ground-state spin Hamiltonian under ultrafast laser excitation. Consequently, a static 64-nm pitch cycloid is obtained as in the proper BiFeO_3_ anisotropy landscape. The AF cycloid dynamics is simulated after application of a 100-fs spin torque pulse mimicking the effect of light-induced spin accumulation at the CoFeB/BiFeO_3_ interface during the ultrafast ferromagnetic demagnetization process. The time evolution of the AF spin texture is assessed during the first 10 ps for the spin torque applied along or perpendicular to the AF cycloidal plane. Figure 4 (A and B) shows the time evolution of each component of magnetic moment averaged over one AF cycloid period. In both cases, a net magnetic moment is induced in the direction of the applied STT pulse, which is the signature of the injected spin angular momentum. The induced moment is halved for an STT applied in the AF cycloidal plane compared to when the STT is perpendicular to it. This can be naively expected, as in this latter case, the STT is equivalent on all spins, whereas in the cycloidal plane, it has a sinusoidal dependence. In both cases, this initial period during the STT pulse is followed by a clear oscillatory behavior at the AF cycloid resonance frequency of 0.68 THz, although with a much larger amplitude when the STT is in the cycloidal plane. The nonzero average magnetic moment induced in the direction of the applied STT shows a highly elliptical oscillation. The resulting overall STT-induced dynamics in the two geometries are fairly different (Fig. 4, C and D). When the STT is along the AF cycloid (Fig. 4C), the spin dynamics is mostly located in the region where the spins are out of plane. The resulting AF dynamics could be defined as the extracyclon $\psi_{1}$ mode (*47*). On the other hand, when STT is perpendicular to the AF cycloidal plane (Fig. 4D), all spins rotate around the induced magnetic moment, leading to an overall shift of the AF cycloid phase, which could be described as the $\phi_{0}$ AF softening cyclon mode (*47*). While a quantitative comparison with the data is beyond the scope of this article, one can conclude that the simulations lead to a response frequency in BiFeO_3_ fully consistent with that measured and a significant anisotropy in the amplitude, again consistent with the experimental observation. This provides further evidence that the measured coherent dynamics in this insulating AF is indeed triggered by STT.

## DISCUSSION

In the context of terahertz spintronics, AFs have a pivotal role in transport and processing of spin information, standing as key components in addressing ultrafast spintronic challenges. However, one crucial issue has remained unresolved: the demonstration of the ultrafast transfer of spin information to AF dynamics, i.e., at picosecond and subpicosecond timescales.

Our study is filling this gap using a smart experimental approach based on cutting-edge time-resolved magneto-optical and birefringence measurements on a prototypical single-domain insulating magnetoelectric AF thin film. The experimental observations have evidenced that terahertz AF dynamics in the insulating AF could be efficiently triggered by the light-induced ultrafast dynamics of an adjacent ferromagnetic layer, playing the role of the ultrafast spin current generator. We conclude that the observed terahertz oscillations were indeed hosted in the AF insulator, thanks to a complex set of angular dependences reported in Fig. 2. Moreover, the experimental observations have also revealed that an incoherent additional spin dissipation channel also plays a strong role. Notably, we could show that this process is as efficient in the gigahertz regime as in the terahertz regime. Although we have demonstrated ultrafast exchanges of spin angular momentum across the CoFeB/BiFeO_3_ interface, multiple types of ultrafast spin currents are likely involved in these ferromagnetic metal/AF insulator bilayers. While the precise mechanisms remain to be fully elucidated, it is reasonable to propose that the dominant process involves the ultrafast buildup of spin accumulation. In this scenario, spin-flip events at the interface exert spin torques on the AF texture, ultimately driving the transport of spin currents through magnonic AF spin excitations. This process constitutes the ultrafast counterpart of spin current injection previously demonstrated in Pt/insulating ferrimagnets [e.g., (*48*)] and Pt/insulating AF systems [e.g., (*11*)] Conceptually, these results are not restricted to magnetoelectric AFs and could be generalized to “pure” insulating AF systems. Therefore, it stands as an important milestone for the implementation of AF insulators in terahertz spintronics devices. Last, in the light of our atomic spin simulations, we elucidate the underlying mechanism driving the terahertz AF dynamics driven by ultrafast spin-transfer torque. We expect that these results will stimulate further investigations on the propagation characteristics of the terahertz spin current through the insulating AF.

## MATERIALS AND METHODS

### Sample preparation

A 66-nm-thick BiFeO_3_ layer was grown by pulsed laser deposition on top of a SrRuO_3_-buffered (011)-oriented DyScO_3_ orthorhombic substrate [(111) pseudocubic orientation] (*43*). This substrate promotes the growth of BiFeO_3_ along its polar axis, resulting in a single ferroelectric domain with a purely out-of-plane polarization pointing downward, as checked with a combination of advanced x-ray diffraction and piezoresponse force microscopy experiments (*43*). Moreover, the anisotropic in-plane epitaxial strain lifts the degeneracy between the AF cycloidal variants. A single AF domain results with its cycloidal propagation direction parallel to the orthorhombic axis of the DyScO_3_ substrate. The AF spin textures of BiFeO_3_ were imaged (Fig. 1B) using a commercial scanning nitrogen-vacancy (NV) magnetometer (ProteusQ, Qnami AG) operated under ambient conditions. The scanning tip is a commercial all-diamond probe with a single NV defect at its apex. It is integrated on a quartz tuning fork (Quantilever MX, Qnami AG) comprising an atomic force microscope combined with a confocal microscope optimized for single NV defect spectroscopy. Subsequently a 5-nm-thick Co_40_Fe_40_B_20_ layer, capped with 2 nm of Al, was simultaneously grown by sputtering on top of the BiFeO_3_ film and on a bare (011) DyScO_3_ substrate for reference.

### Time-resolved magneto-optical experiments

The laser pulses are generated by a 1-kHz Ti:sapphire amplifier with a pulse duration of 65 fs. To perform thorough angular dependences of the probe and pump polarizations, half waveplates and polarizers were added in the beams, slightly modifying the time resolution. The pump (800-nm wavelength) is focused on a 300-μm spot with a 60° incident angle. The probe (400-nm wavelength) is focused on a 22-μm spot at a 44° incidence angle with respect to the sample plane. After reflection on the sample, the ultrafast change in light characteristics is measured by a standard polarization bridge. The probe fluence is about 75 μJ/cm^2^, while the pump fluence was varied between 1.5 and 9 mJ/cm^2^.

To extract the relevant parameters, the experimental data are fitted with the following phenomenological model

$$m\left(t\right)=\Theta \left(t\right)⊗H\left(t\right)\times \left(A_{D}e^{-\frac{t}{\tau_{D}}}+A_{R}e^{-\frac{t}{\tau_{R}}}+\cdots \right)$$

where m(t) is the magnetization, H(t) is the Heaviside step function, and several exponential terms are assessing the characteristics of the various demagnetization and remagnetization processes. The whole magnetization dynamics profile is convoluted by 2 Gaussian curves [Θ(t)] to reproduce the effect of the experimental time resolution due to both the pump and probe temporal profiles.

### Atomic spin dynamics simulations

On a general viewpoint, simulating AF properties requires to consider individual spins. Atomic spin dynamics simulations have therefore been carried out using a GPU-based homemade code [see (*44*)]. The ground state of our [111]-oriented BiFeO_3_ is found to be a 64-nm pitched single AF cycloid along the [1-10] direction. Here, we consider a 64 nm–by–1.94 nm–by–1.38 nm simulated area, in a frame defined by the [1-10], [11-2], and [111] directions, with periodic boundary conditions in all directions. This reproduces a BiFeO_3_ layer hosting a single cycloid with the ferroelectric polarization along the [111] direction. To simulate the effect of an ultrafast spin current, damping-like STT is applied for the first 100 fs on the whole simulated area. The STT is expected to be applied only on the few first nanometers from the interface of our 66-nm-thick BiFeO_3_ layer. Nonetheless, the simulations should capture the main features of the STT-induced dynamics of the AF cycloid. More modeling details can be found in Li *et al.* (*46*). Besides, general details of the BiFeO_3_ Hamiltonian used for the simulations and the values of the various quantities have been reported in Fishman *et al.* (*49*) and Nagel *et al.* (*50*). The pump wavelength (1.55 eV) being well below the BiFeO_3_ bandgap (about 3 eV), we consider that the BiFeO_3_ keeps its ground-state spin Hamiltonian under ultrafast laser excitation of a few millijoules per square centimeter.

## Supplementary Materials

### This PDF file includes:

Notes S1 to S6 Figs. S1 to S15 Table S1 References
