# Supplementary material for: Terahertz antiferromagnetic dynamics induced by ultrafast spin currents
Source: Sci Adv. 2025 Nov 7;11(45):eadx1107. doi: 10.1126/sciadv.adx1107 (PMC12594165; doi:10.1126/sciadv.adx1107)
Supplement: Supplementary file 1 — Notes S1 to S6 Figs. S1 to S15 Table S1 References [file sciadv.adx1107_sm.pdf]

Science Advances
AAAS

Supplementary Materials for

Terahertz antiferromagnetic dynamics induced by ultrafast spin currents

Sanjay René *et al.*

Corresponding author: Jean-Yves Chauleau, jean-yves.chauleau@cea.fr

Sci. Adv. **11**, eadx1107 (2025)
DOI: 10.1126/sciadv.adx1107

This PDF file includes:

Notes S1 to S6
Figs. S1 to S15
Table S1
References

Supplementary Note 1: Description of static MOKE measurements for TR-MOKE calibration

In order to correlate the raw TR-MOKE signal to a change in magnetization amplitude, we perform a static MOKE calibration measurement prior to any time-resolved measurement. Without pump, the probe signal is acquired while the applied magnetic field is rotated by 360° (Figure S1). Note that in this case, the probe beam is modulated by the optical chopper.

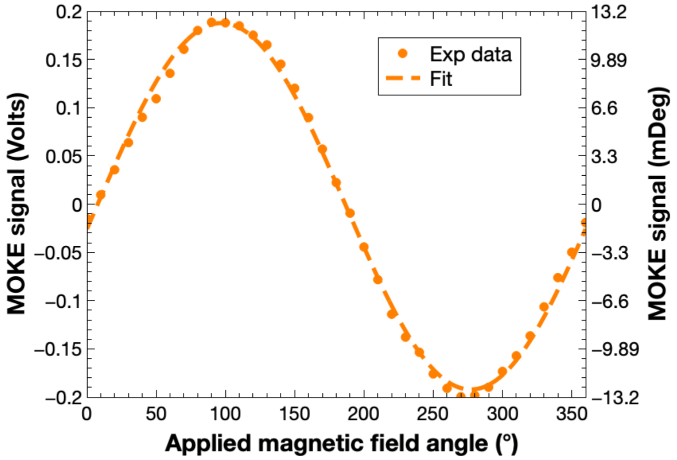

Fig. S1: Example of static MOKE calibration*. Measurement performed on the CoFeB/BiFeO$_3$ sample for the p-polarized probe given in Volts (raw signal) and after in mDeg (after calibration of the polarization bridge).*

Supplementary Note 2: Pump light absorption:

In this study, it is important to quantitatively compare the ultrafast dynamics of the CoFeB/BiFeO$_3$ and the CoFeB reference layer. The sole knowledge of the pump fluence at the sample surface is not enough. The absorbed power of the pump light in the CoFeB layer is required and can significantly change depending on the layer stacking. Its precise measurements in stack of thin layers grown on top of a thick substrate is rather complicated. However, using a transfer matrix method (TMM), one can calculate, with rather good confidence, the expected landscape of the absorbed power. The code developed by S. Byrnes (https://pypi.org/project/tmm/) has been used for this purpose including the complex refractive indexes ($\tilde{n} = n + i \cdot k$) of all involved materials (see Supplementary Table 1) from the literature. The BiFeO$_3$ index has been taken from ref 51 where it has been measured for thin BiFeO$_3$ epitaxial layers on various substrates, including DyScO$_3$.

Table S1 : list of refractive indexes used in the TMM calculations.

	n	k
AlO_x	1.7601	0.0
$Co_{40}Fe_{40}B_{20}$	3.0	3.0
$BiFeO_3$	2.784	0.0
$SrRuO_3$	2.073	1.97
$DyScO_3$	2.0	0.0

Supplementary Figure S2 displays the calculated absorbed power profiles across the two kinds of stacks for the set of refractive indexes given in Table 1. The main conclusion is that for a given incident light fluence, about twice less power is absorbed in the CoFeB layer in the case of the CoFeB/BiFeO$_3$ multilayer than for the reference where the CoFeB is directly on top of the DyScO$_3$ substrate.

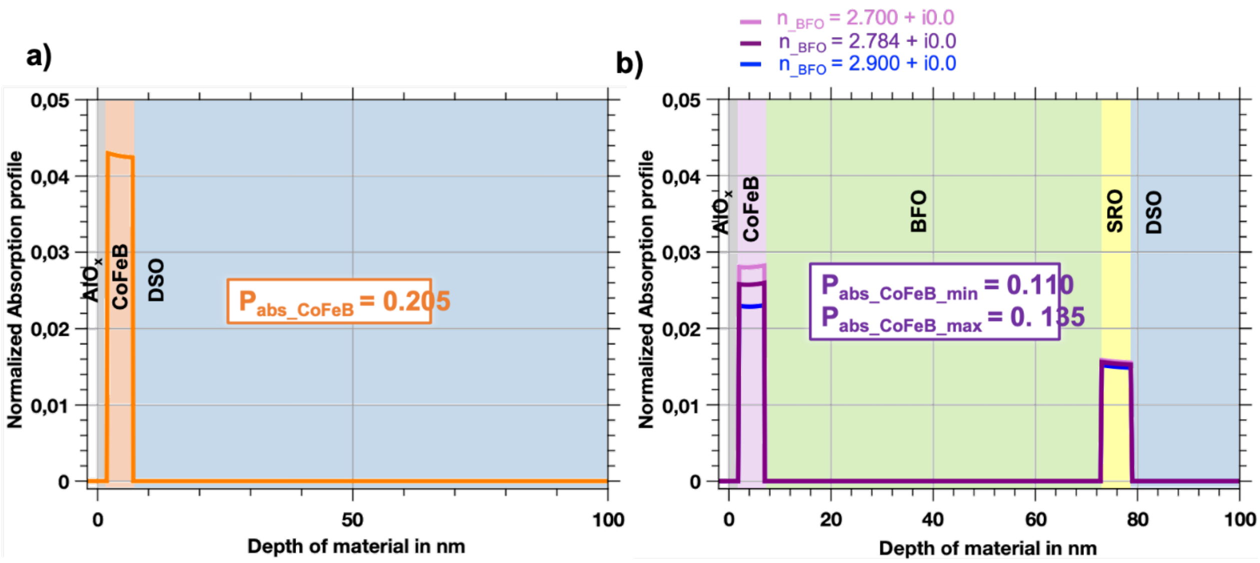

Fig. S2: Light absorption in multilayers. *Normalized absorption profiles for a) the CoFeB reference layer and b) the CoFeB/BiFeO_3 bilayer.*

Supplementary Note 3: Ferromagnetic resonance study (FMR):

Ferromagnetic resonance (FMR - Supplementary Figure S3) was measured in a standard broadband experimental setup where the samples are positioned on a stripline which generates the GHz pumping magnetic field. In our experimental configuration, the excitation frequency is kept constant, typically ranging from 4 to 14 GHz, while the applied magnetic field is swept across the resonance condition. A low frequency modulated magnetic field is superimposed in order to allow a lock-in detection and therefore an increased experimental sensitivity. As a result of a lock-in demodulation, the measured signal is the derivative of the absorption spectrum with respect to the applied magnetic field (Supplementary Figure S3). The obtained experimental data are fitted by the derivative of a standard asymmetric Lorentzian function allowing us to extract the two important quantities, namely: the resonance field (H_{res}) and the linewidth (ΔH).

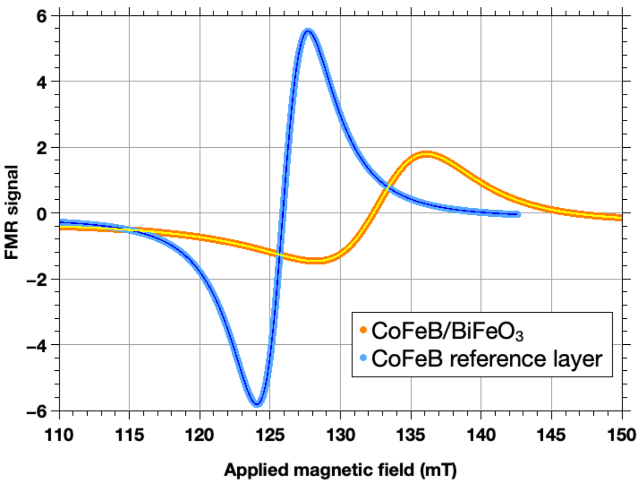

Fig. S3: Typical FMR derivative absorption spectra. *Measurements performed at an excitation frequency of 8 GHz for the CoFeB/BiFeO_3 (purple) and CoFeB reference (orange) layers with their respective fits.*

It is to be noted that in the presence of a uniaxial in-plane magnetic anisotropy (H_K), the relationship, known as Kittel's formula (ref 52), between the excitation frequency (f), the resonance field and the internal magnetic energies, can be written in the following way:

$$\frac{2\pi f}{\mu_0 \gamma} = \sqrt{(H_{\mathrm{res}} + H_K)(H_{\mathrm{res}} + H_K + M_S)}$$

Where M_S is the saturation magnetization in the absence of out-of-plane anisotropy, γ the gyromagnetic ratio and μ_0 the vacuum permittivity, and $H_K = {}^{-2K}/_{\mu_0 M_S}$.

For the reference layer, a small uniaxial anisotropy ($\mu_0 H_K \sim 2 - 3$ mT) is observed whereas the contact with the AF layer in CoFeB/BiFeO_3 significantly increases the anisotropy ($\mu_0 H_K \sim 11 - 16$ mT). In both cases, the easy axis is perpendicular to the DyScO_3 a-axis, i.e., perpendicular to the AF cycloidal plane in the case of the CoFeB/BiFeO_3 bilayer. This enhancement of the magneto-crystalline anisotropy is a signature of the exchange coupling often reported at the ferromagnet/AF interface. Note that $\mu_0 M_S \sim 1.13 \pm 0.01\, T$ can be estimated.

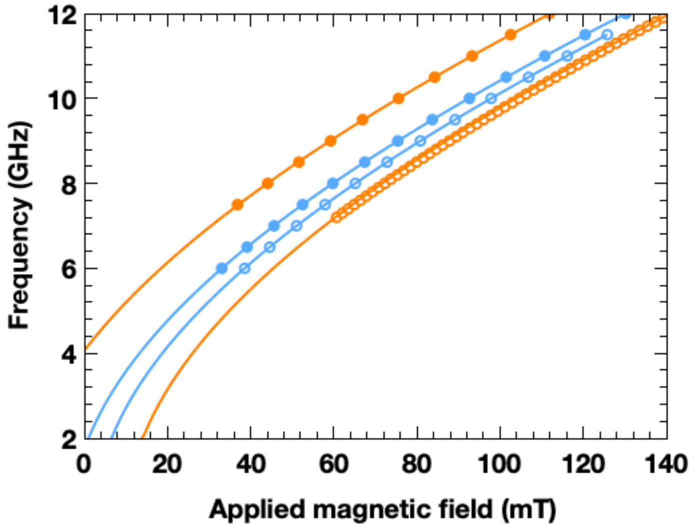

Fig. S4: Field dependence of the resonance frequency. *For each sample, two configurations are considered with the external magnetic field applied either along (opened dots) or perpendicular (full dots) to the a-axis of the $DyScO_3$ substrate (also, parallel to the propagation direction the $BiFeO_3$ cycloid).*

Supplementary Note 4: Additional time-resolved MOKE measurements:

The ultrafast dynamics of the CoFeB/$BiFeO_3$ bilayer is assessed with the in-plane magnetization of the CoFeB layer set either perpendicularly or parallel to the AF cycloidal plane. This can be achieved either by rotating the sample by 90° in a fixed DC magnetic field or by rotating the field to set the CoFeB magnetization transversally to the incident probe pulse plane. The former geometry is that of the traditional longitudinal MOKE measurement. The latter configuration only measures the transverse MOKE leading to a much smaller effect on the reflected light amplitude where the ultrafast demagnetization in the CoFeB layer is not visible and the main contribution, at short timescales, originates from the $BiFeO_3$ birefringence. This is also highly dependent on the relative direction between the probe/pump light propagations and the crystal lattice, so one cannot rely on rotating the sample to isolate the STT-induced dynamics per se. On the other hand, unlike MOKE from the CoFeB magnetization dynamics (Fig. S6), birefringence is expected to sensitively depend on the polarization state of the probe (either p-polarized or s-polarized), suggesting that any pronounced dependence on the probe polarization would be the signature of the internal dynamics of $BiFeO_3$. Note that GHz FMR oscillations are observed for both CoFeB and CoFeB/$BiFeO_3$ samples (Fig. S7).

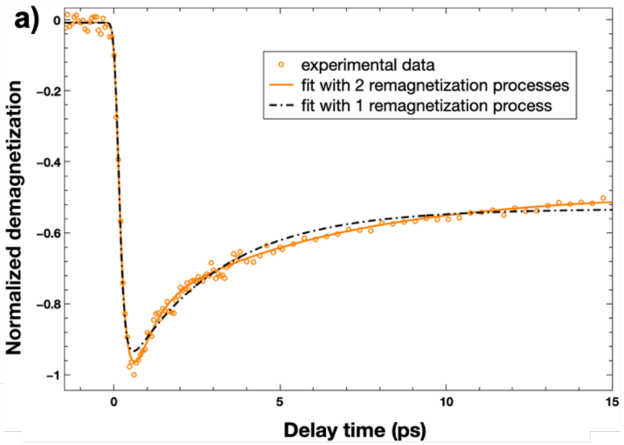

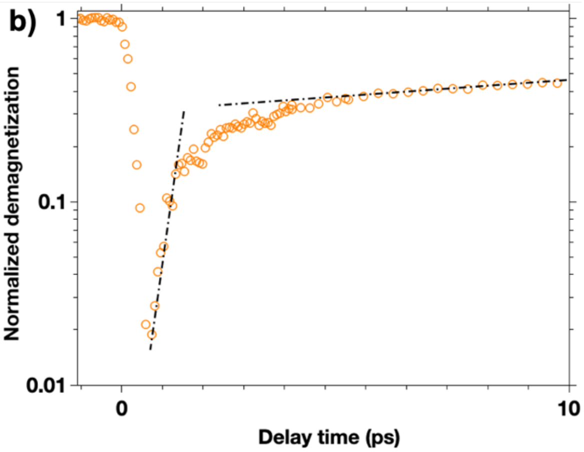

Fig. S5: Analysis of the CoFeB/$BiFeO_3$ ultrafast dynamics *a) Comparison of the CoFeB/$BiFeO_3$ ultrafast dynamics (orange dots) with fits using either 1 remagnetization process (black dotted line) with a characteristic time of 2.3 ps, 2 remagnetization processes (orange line) with characteristic times of 0.5 and 6 ps. b) CoFeB/$BiFeO_3$ bilayer normalized ultrafast magnetization dynamics displayed in logarithmic scale. Dashed lines are guide for the eye.*

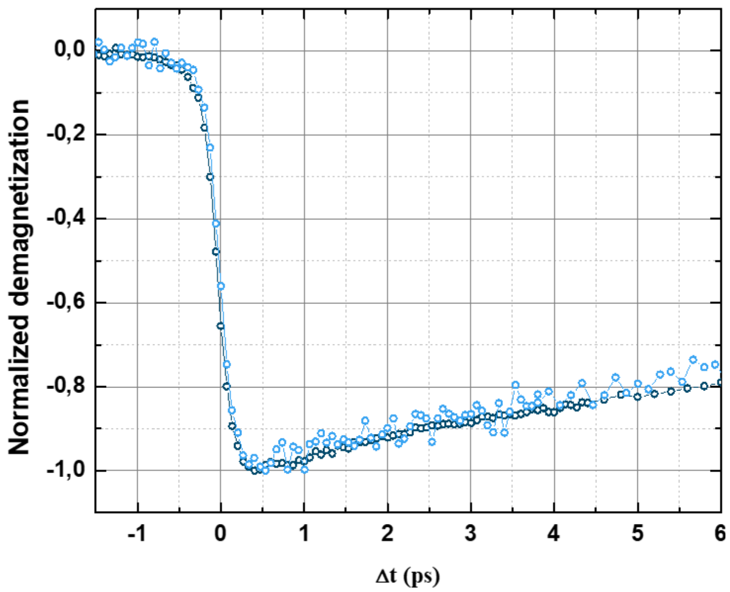

Fig. S6: Comparison of the ultrafast dynamics of the CoFeB reference layer. *Measurements performed for a p-polarized (black) and s-polarized (blue) 400 nm probe.*

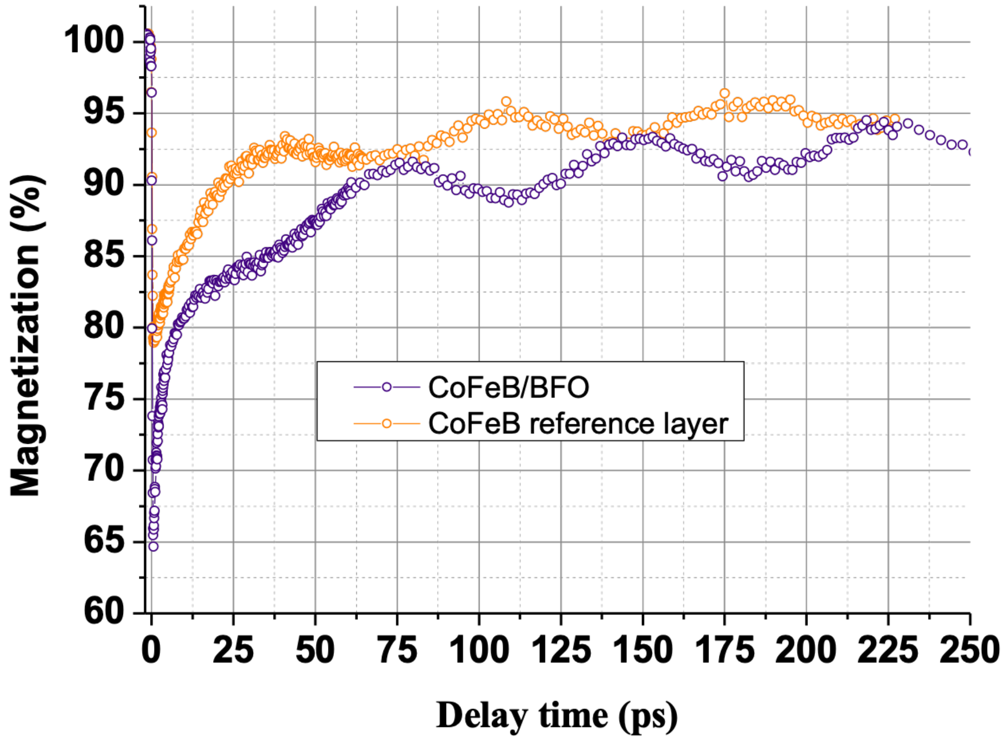

Fig. S7: Magnetization dynamics at long timescales. *Tr-MOKE measurements for the CoFeB/$BiFeO_3$ (purple) and CoFeB (orange) layers at long timescales. Note that another oscillation is also visible at about 12 GHz, which corresponds to the lower frequency ferromagnetic resonance measured in the CoFeB layer.*

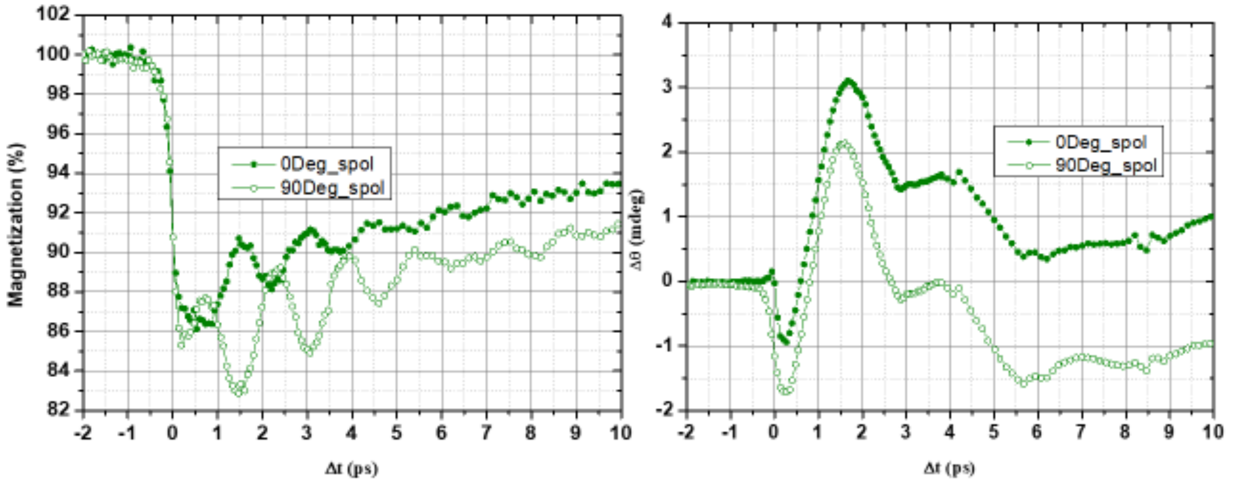

***Fig. S8: Tr-MOKE measurements on the CoFeB/BiFeO$_3$ multilayer for a s-polarized probe**. Standard ultrafast magnetization dynamics (left panel) are obtained by performing the difference between the two opposite applied magnetic field directions. This usual procedure allows to remove the pure optical contributions in the observed light-induced dynamics. On the contrary, summing them (right panel) leads to the observation of the optical dynamics without contributions from the ultrafast magnetization dynamics. Importantly, the 0.6 THz is not visible in the optical part which proves that the relevant signal does not come from a phononic contribution. This strengthens the role of the ultrafast STT in the triggered dynamics.*

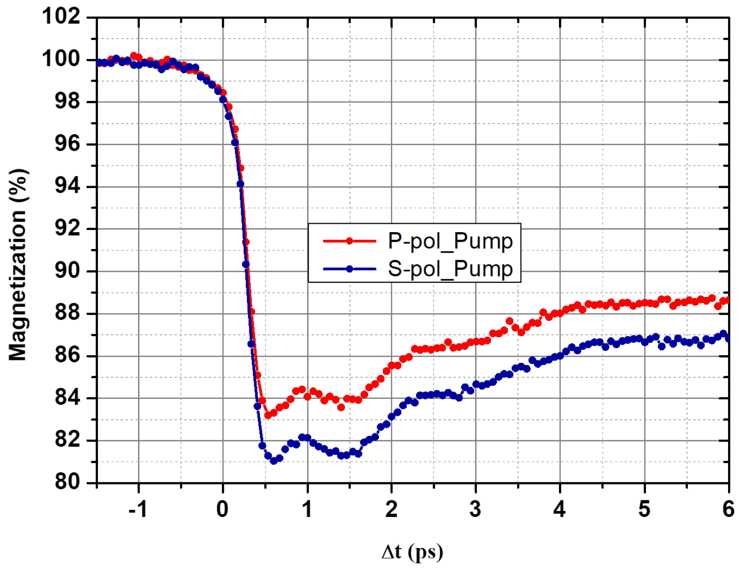

***Fig. S9: Comparison of the measured ultrafast dynamics in the CoFeB/BiFeO$_3$ bilayer**. Measurements performed for two pump polarization states: p-polarized (red) and s-polarized (blue). Except for a slight difference in amplitude, the two dynamical behaviors are identical. This emphasizes that the triggered THz dynamics in the BiFeO$_3$ layer is not due to direct optical pumping.*

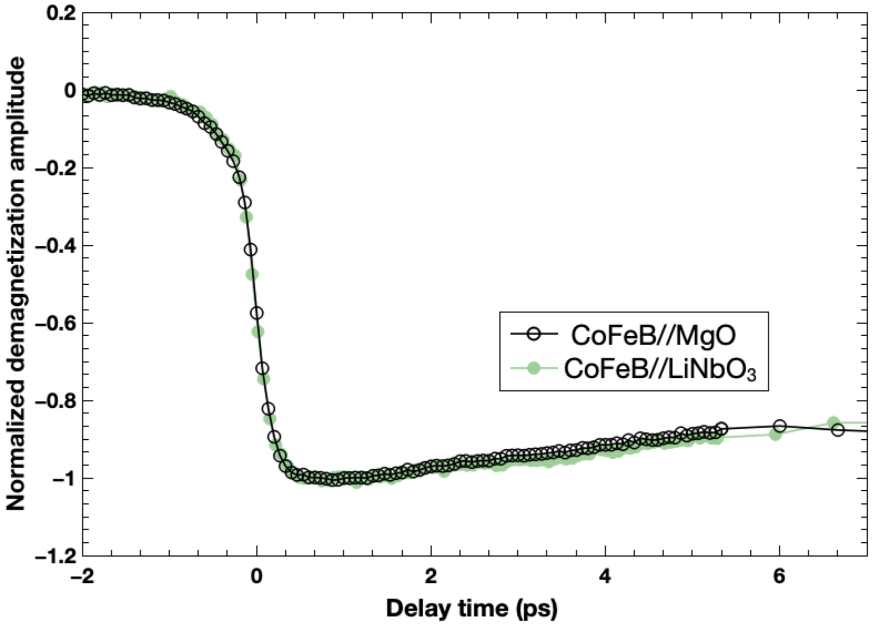

Fig. S10: Comparison of the CoFeB ultrafast dynamics when grown on either a MgO (001) substrate or on a ferroelectric $LiNbO_3$ (111) substrate*. Note that the $LiNbO_3$ has the same rhombohedral 3m symmetry as $BiFeO_3$. The dynamics overlap perfectly, thus ruling out ferroelectricity as a potential cause for the observed fast remagnetization process.*

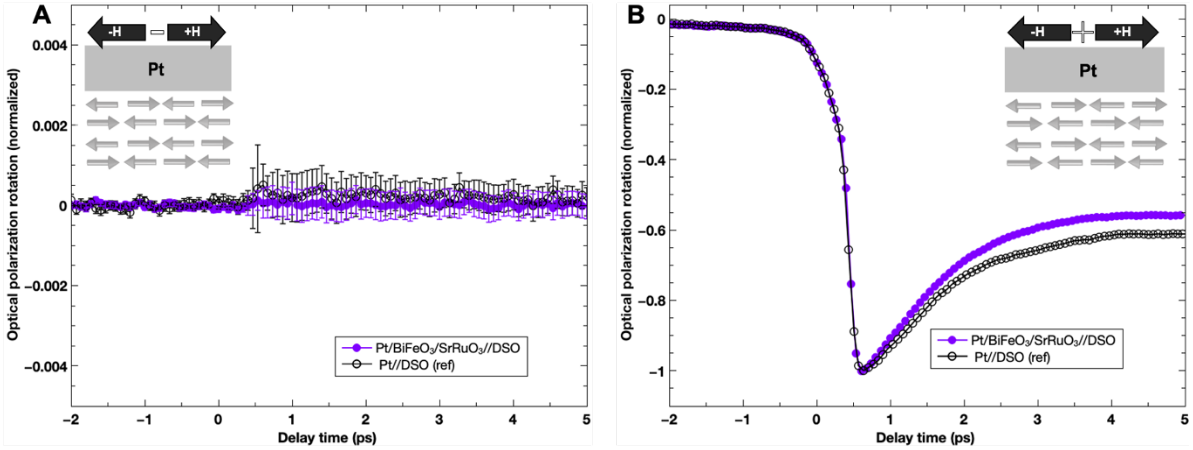

Fig S11: Ultrafast light induced dynamics of a Pt (5nm)/$BiFeO_3$(20nm)/$SrRuO_3$(15nm)//$DyScO_3$ sample*. Difference (A) and sum (B) of the two signals obtained for two opposite directions of the external applied field. These measurements have been performed for a S polarized probe pulse with an incident propagation plane perpendicular to AF cycloid plane (DSO a-axis in case of the reference sample – black opened dots.).*

Supplementary Note 5: $BiFeO_3$ atomic spin dynamics simulations and dynamical polarization

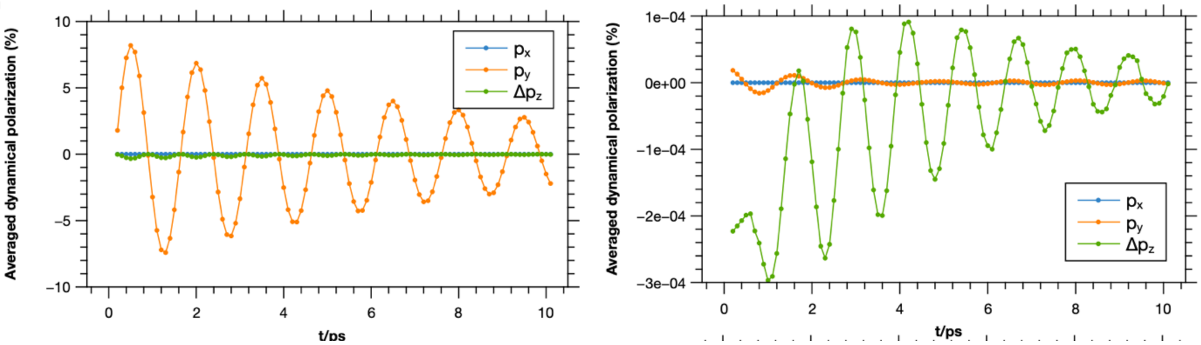

Fig. S12: Atomistic spin dynamics simulations. *Dynamical electrical polarization emerging from the STT-induced dynamics of the AF cycloid obtained from atomic spin simulations for the STT applied in (left panel) and perpendicular to (right panel) the AF cycloidal plane. The x, y components are along and perpendicular to the AF cycloid k-vector, respectively, while the z-component is along the ferroelectric polarization direction (111).*

The observed dynamical birefringence is related to an overall change of the electrical polarization state in the $BiFeO_3$ layer. A complete assessment of this latter is challenging. However, from the full simulated spin texture dynamics, one can partly grasp it by evaluating the associated averaged dipolar moment $\vec{p}$ for each time step using the usual magneto-electric coupling term:

$$\vec{p} = \frac{\sum_{<i,j>} \vec{e}_{ij} \times (\vec{S}_i \times \vec{S}_j)}{N}$$

where $\vec{S}_i$ and $\vec{S}_j$ are local atomic spins, N the total number of spins and $\vec{e}_{ij} = (\vec{r}_j - \vec{r}_i)/\|\vec{r}_j - \vec{r}_i\|$ a unit vector. These simulations prove that a non-zero dynamical polarization can emerge from the STT-induced AF dynamics at the same frequency.

Supplementary Note 6: CoFeB/$BiFeO_3$ characterization

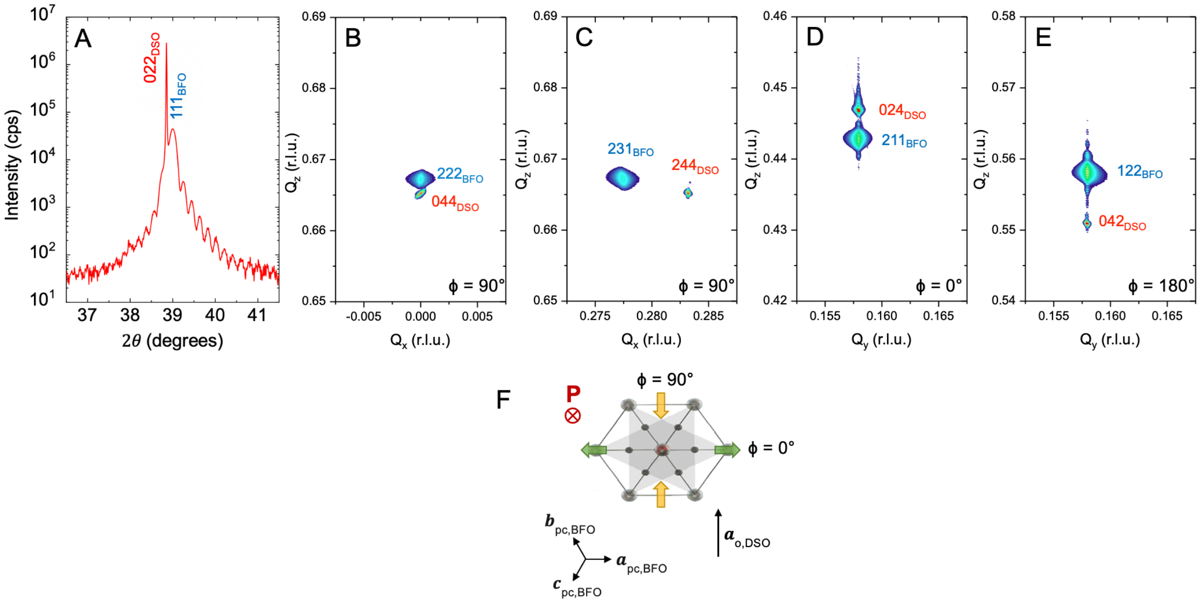

Fig. S13. $BiFeO_3$ layer structural characterizations*. X-ray diffraction characterization of a $BiFeO_3(111)$ / $SrRuO_3$ // $DyScO_3(011)o$ sample. Here the thicknesses of $BiFeO_3$ and $SrRuO_3$ are 50 nm and 5 nm, respectively. A, 2θ-ω X-ray diffraction pattern showing Laue fringes for the BiFeO3 film. B-E, Reciprocal space maps around the (044)o, (244)o, (024)o, and (042)o peaks of $DyScO_3$. F, Sketch of the distorted hexagonal lattice of $BiFeO_3(111)$ under anisotropic strain with $DyScO_3$. The film is under tensile strain perpendicularly to the orthorhombic a_o axis of $DyScO_3$ and compressive strain along a_o.*

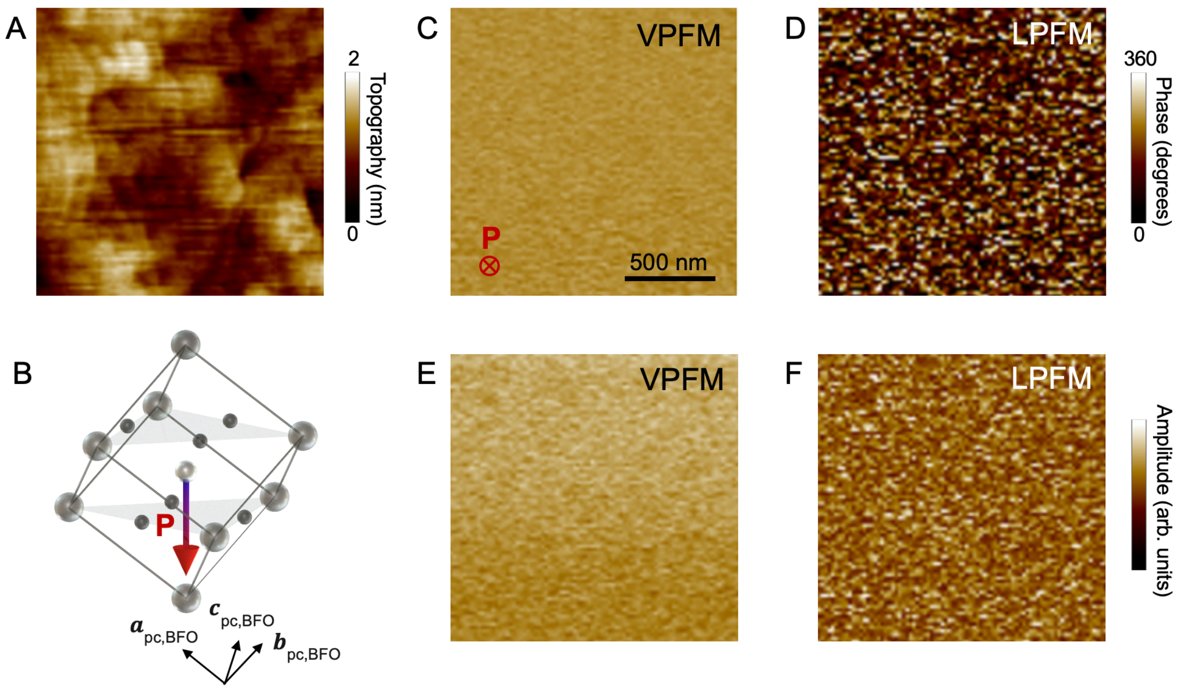

Fig. S14. Ferroelectric domain characterization of the $BiFeO_3(111)$ / $SrRuO_3$ // $DyScO_3(011)o$ sample using piezoresponse force microscopy (PFM). *A, Topography. B, Sketch of the single domain ferroelectric structure. C, Vertical PFM (VPFM) phase. D, Lateral PFM (LPFM) phase. E, VPFM amplitude. F, LPFM amplitude.*

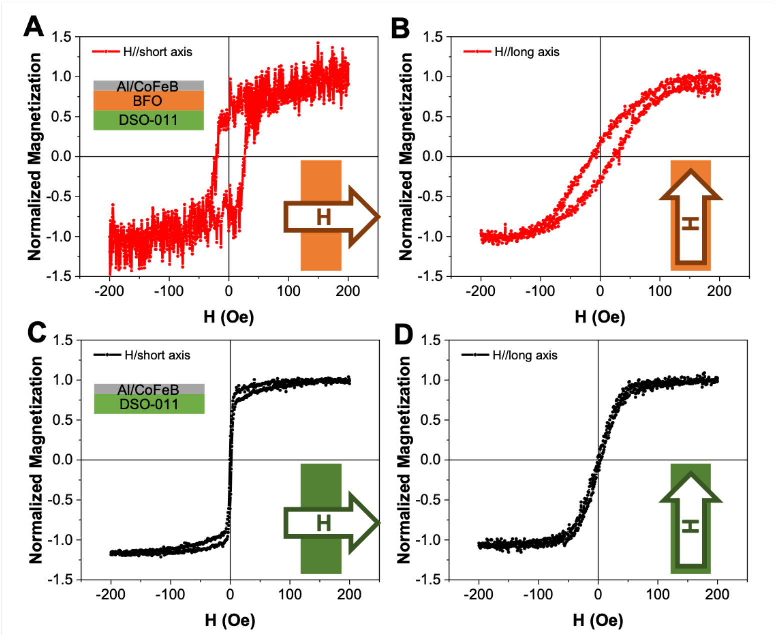

Fig. S15. Alternating Gradient Field Magnetometry (AGFM). *Measurements performed on CoFeB/$BiFeO_3$ (A & B) and CoFeB (C and D) layers. While no exchange bias is observed, the effect of exchange anisotropy is clearly visible on the CoFeB coercive fields for the bilayer, which corroborate the FMR analysis. Note that magnetization amplitudes have been normalized as a large paramagnetic background prevents a thorough quantitative analysis.*

REFERENCES AND NOTES

1. L. NeéL, Antiferromagnetism and ferrimagnetism. *Proc. Phys. Soc. A* **65**, 869 (1952).

2. V. Baltz, A. Manchon, M. Tsoi, T. Moriyama, T. Ono, Y. Tserkovnyak, Antiferromagnetic spintronics. *Rev. Mod. Phys.* **90**, 015005 (2018).

3. M. B. Jungfleisch, W. Zhang, A. Hoffmann, Perspectives of antiferromagnetic spintronics. *Phys. Lett. A* **382**, 865–871 (2018).

4. C. Kittel, Theory of antiferromagnetic resonance. *Phys. Rev.* **82**, 565 (1951).

5. F. Keffer, C. Kittel, Theory of antiferromagnetic resonance. *Phys. Rev.* **85**, 329 (1952).

6. A. J. Sievers, M. Tinkham, Far infrared antiferromagnetic resonance in MnO and NiO. *Phys. Rev.* **129**, 1566 (1963).

7. T. Kampfrath, A. Sell, G. Klatt, A. Pashkin, S. Mährlein, T. Dekorsy, M. Wolf, M. Fiebig, A. Leitenstorfer, R. Huber, Coherent terahertz control of antiferromagnetic spin waves. *Nat. Photon.* **5**, 31–34 (2011).

8. H. Wang, C. Du, P. C. Hammel, F. Yang, Antiferromagnonic spin transport from $Y_3Fe_5O_{12}$ into NiO. *Phys. Rev. Lett.* **113**, 097202 (2014).

9. C. Hahn, G. de Loubens, V. V. Naletov, J. Ben Youssef, O. Klein, M. Viret, Conduction of spin currents through insulating antiferromagnetic oxides. *Euro. Phys. Lett.* **108**, 57005 (2014).

10. W. Lin, K. Chen, S. Zhang, C. L. Chien, Enhancement of thermally injected spin current through an antiferromagnetic insulator. *Phys. Rev. Lett.* **116**, 186601 (2016).

11. R. Lebrun, Tunable long-distance spin transport in a crystalline antiferromagnetic iron oxide. *Nature* **561**, 222–225 (2018).

12. A. Ross, R. Lebrun, O. Gomonay, D. A. Grave, A. Kay, L. Baldrati, S. Becker, A. Qaiumzadeh, C. Ulloa, G. Jakob, F. Kronast, J. Sinova, R. Duine, A. Brataas, A. Rothschild,

M. Kläui, Propagation length of antiferromagnetic magnons governed by domain configurations. *Nano Lett.* **20**, 306–313 (2020).

13. R. Khymyn, I. Lisenkov, V. S. Tiberkevich, A. N. Slavin, B. A. Ivanov, Transformation of spin current by antiferromagnetic insulators. *Phys. Rev. B* **93**, 224421 (2016).

14. J. Han, P. Zhang, Z. Bi, Y. Fan, T. S. Safi, J. Xiang, J. Finley, L. Fu, R. Cheng, L. Liu, Birefringence-like spin transport via linearly polarized antiferromagnetic magnons. *Nat. Nanotechnol.* **15**, 563–568 (2020).

15. A. Brataas, Y. Tserkovnyak, G. E. W. Bauer, P. J. Kelly, Spin pumping and spin transfer, in *Spin Current*, S. Maekawa, S. O. Valenzuela, E. Saitoh, T. Kimura, Eds., (Oxford Univ. Press), pp. 87–135 (2017).

16. R. Cheng, J. Xiao, Q. Niu, A. Brataas, Spin pumping and spin-transfer torques in antiferromagnets. *Phys. Rev. Lett.* **113**, 057601 (2014).

17. J. Li, C. B. Wilson, R. Cheng, M. Lohmann, M. Kavand, W. Yuan, M. Aldosary, N. Agladze, P. Wei, M. S. Sherwin, J. Shi, Spin current from sub-terahertz-generated antiferromagnetic magnons. *Nature* **578**, 70–74 (2020).

18. P. Vaidya, S. A. Morley, J. van Tol, Y. Liu, R. Cheng, A. Brataas, D. Lederman, E. del Barco, Subterahertz spin pumping from an insulating antiferromagnet. *Science* **368**, 160–165 (2020).

19. E. Rongione, O. Gueckstock, M. Mattern, O. Gomonay, H. Meer, C. Schmitt, R. Ramos, T. Kikkawa, M. Mičica, E. Saitoh, J. Sinova, H. Jaffrès, J. Mangeney, S. T. B. Goennenwein, S. Geprägs, T. Kampfrath, M. Kläui, M. Bargheer, T. S. Seifert, S. Dhillon, R. Lebrun, Emission of coherent THz magnons in an antiferromagnetic insulator triggered by ultrafast spin–phonon interactions. *Nat. Commun.* **14**, 1818 (2023).

20. E. Beaurepaire, J.-C. Merle, A. Daunois, J.-Y. Bigot, Ultrafast spin dynamics in ferromagnetic nickel. *Phys. Rev. Lett.* **76**, 4250 (1996).

21. J.-Y. Bigot, M. Vomir, Ultrafast magnetization dynamics of nanostructures. *Ann. Phys.* **525**, 2–30 (2013).

22. A. Kirilyuk, A. V. Kimel, T. Rasing, Ultrafast optical manipulation of magnetic order. *Rev. Mod. Phys.* **82**, 2731 (2010).

23. G. Malinowski, N. Bergeard, M. Hehn, S. Mangin, Hot-electron transport and ultrafast magnetization dynamics in magnetic multilayers and nanostructures following femtosecond laser pulse excitation. *Eur. Phys. J. B* **91**, 98 (2018).

24. J. Walowski, M. Münzenberg, Perspective: Ultrafast magnetism and THz spintronics. *J. Appl. Phys.* **120**, 140901 (2016).

25. B. Koopmans, G. Malinowski, F. D. Longa, D. Steiauf, M. Fähnle, T. Roth, M. Cinchetti, M. Aeschlimann, Explaining the paradoxical diversity of ultrafast laser-induced demagnetization. *Nat. Mater.* **9**, 259–265 (2010).

26. J. K. Dewhurst, P. Elliott, S. Shallcross, E. K. U. Gross, S. Sharma, Laser-induced intersite spin transfer. *Nano Lett.* **18**, 1842–1848 (2018).

27. F. Siegrist, J. A. Gessner, M. Ossiander, C. Denker, Y.-P. Chang, M. C. Schröder, A. Guggenmos, Y. Cui, J. Walowski, U. Martens, J. K. Dewhurst, U. Kleineberg, M. Münzenberg, S. Sharma, M. Schultze, Light-wave dynamic control of magnetism. *Nature* **571**, 240–244 (2019).

28. G. Malinowski, F. Dalla Longa, J. H. H. Rietjens, P. V. Paluskar, R. Huijink, H. J. M. Swagten, B. Koopmans, Control of speed and efficiency of ultrafast demagnetization by direct transfer of spin angular momentum. *Nat. Phys.* **4**, 855–858 (2008).

29. M. Battiato, K. Carva, P. M. Oppeneer, Superdiffusive spin transport as a mechanism of ultrafast demagnetization. *Phys. Rev. Lett.* **105**, 027203 (2010).

30. A. Melnikov, I. Razdolski, T. O. Wehling, E. T. Papaioannou, V. Roddatis, P. Fumagalli, O. Aktsipetrov, A. I. Lichtenstein, U. Bovensiepen, Ultrafast transport of laser-excited spin-polarized carriers in Au/Fe/MgO(001). *Phys. Rev. Lett.* **107**, 076601 (2011).

31. A. Alekhin, I. Razdolski, N. Ilin, J. P. Meyburg, D. Diesing, V. Roddatis, I. Rungger, M. Stamenova, S. Sanvito, U. Bovensiepen, A. Melnikov, Femtosecond spin current pulses

generated by the nonthermal spin-dependent seebeck effect and interacting with ferromagnets in spin valves. *Phys. Rev. Lett.* **119**, 017202 (2017).

32. M. Battiato, K. Carva, P. M. Oppeneer, Theory of laser-induced ultrafast superdiffusive spin transport in layered heterostructures. *Phys. Rev. B* **86**, 024404 (2012).

33. E. G. Tveten, A. Brataas, Y. Tserkovnyak, Electron-magnon scattering in magnetic heterostructures far out of equilibrium. *Phys. Rev. B* **92**, 180412 (2015).

34. M. Beens, R. A. Duine, B. Koopmans, $s - d$ Model for local and nonlocal spin dynamics in laser- excited magnetic heterostructures. *Phys. Rev. B* **102**, 054442 (2020).

35. I. Razdolski, A. Alekhin, N. Ilin, J. P. Meyburg, V. Roddatis, D. Diesing, U. Bovensiepen, A. Melnikov, Nanoscale interface confinement of ultrafast spin transfer torque driving non-uniform spin dynamics. *Nat. Commun.* **8**, 15007 (2017).

36. T. Kampfrath, M. Battiato, P. Maldonado, G. Eilers, J. Nötzold, S. Mährlein, V. Zbarsky, F. Freimuth, Y. Mokrousov, S. Blügel, M. Wolf, I. Radu, P. M. Oppeneer, M. Münzenberg, Terahertz spin current pulses controlled by magnetic heterostructures. *Nat. Nanotechnol.* **8**, 256–260 (2013).

37. T. Seifert, S. Jaiswal, U. Martens, J. Hannegan, L. Braun, P. Maldonado, F. Freimuth, A. Kronenberg, J. Henrizi, I. Radu, E. Beaurepaire, Y. Mokrousov, P. M. Oppeneer, M. Jourdan, G. Jakob, D. Turchinovich, L. M. Hayden, M. Wolf, M. Münzenberg, M. Kläui, T. Kampfrath, Efficient metallic spintronic emitters of ultrabroadband terahertz radiation. *Nat. Photon.* **10**, 483–488 (2016).

38. E. T. Papaioannou, R. Beigang, THz spintronic emitters: A review on achievements and future challenges. *Nanophotonics* **10**, 1243 (2021).

39. S. R. Burns, O. Paull, J. Juraszek, V. Nagarajan, D. Sando, The experimentalist's guide to the cycloid, or noncollinear antiferromagnetism in epitaxial $BiFeO_3$. *Adv. Mater.* **32**, 2003711 (2020).

40. M. Cazayous, Y. Gallais, A. Sacuto, R. de Sousa, D. Lebeugle, D. Colson, Possible observation of cycloidal electromagnons in $BiFeO_3$. *Phys. Rev. Lett.* **101**, 037601 (2008).

41. D. Sando, A. Agbelele, D. Rahmedov, J. Liu, P. Rovillain, C. Toulouse, I. C. Infante, A. P. Pyatakov, S. Fusil, E. Jacquet, C. Carrétéro, C. Deranlot, S. Lisenkov, D. Wang, A. Sacuto, J. Juraszek, A. K. Zvezdin, L. Bellaiche, B. Dkhil, A. Barthélémy, M. Bibes, Crafting the magnonic and spintronic response of $BiFeO_3$ films by epitaxial strain. *Nat. Mater.* **12**, 641–646 (2013).

42. A. Haykal, J. Fischer, W. Akhtar, J.-Y. Chauleau, D. Sando, A. Finco, F. Godel, Y. A. Birkhölzer, C. Carrétéro, N. Jaouen, M. Bibes, M. Viret, S. Fusil, V. Jacques, V. Garcia, Antiferromagnetic textures in $BiFeO_3$ controlled by strain and electric field. *Nat. Commun.* **11**, 1704 (2020).

43. P. Dufour, A. Abdelsamie, J. Fischer, A. Finco, A. Haykal, M. F. Sarott, S. Varotto, C. Carrétéro, S. Collin, F. Godel, N. Jaouen, M. Viret, M. Trassin, K. Bouzehouane, V. Jacques, J.-Y. Chauleau, S. Fusil, V. Garcia, Onset of multiferroicity in prototypical single-spin cycloid $BiFeO_3$ thin films. *Nano Lett.* **23**, 9073 (2023).

44. L. Frangou, S. Oyarzún, S. Auffret, L. Vila, S. Gambarelli, V. Baltz, Enhanced spin pumping efficiency in antiferromagnetic IrMn thin films around the magnetic phase transition. *Phys. Rev. Lett.* **116**, 077203 (2016).

45. P. Khan, M. Kanamaru, K. Matsumoto, T. Ito, T. Satoh, Ultrafast light-driven simultaneous excitation of coherent terahertz magnons and phonons in multiferroic $BiFeO_3$. *Phys. Rev. B* **101**, 134413 (2020).

46. Z. Li, T. Chirac, J. Tranchida, V. Garcia, S. Fusil, V. Jacques, J.-Y. Chauleau, M. Viret, Multiferroic skyrmions in $BiFeO_3$. *Phys. Rev. Res.* **5**, 043109 (2023).

47. R. De Sousa, J. E. Moore, Optical coupling to spin waves in the cycloidal multiferroic $BiFeO_3$. *Phys. Rev. B* **77**, 012406 (2008).

48. L. J. Cornelissen, J. Liu, R. A. Duine, J. B. Youssef, B. J. Van Wees, Long-distance transport of magnon spin information in a magnetic insulator at room temperature. *Nat. Phys.* **11**, 1022–1026 (2015).

49. R. S. Fishman, The microscopic model of $BiFeO_3$. *Phys. B. Condens. Matter* **536**, 115–117 (2018).

50. U. Nagel, R. S. Fishman, T. Katuwal, H. Engelkamp, D. Talbayev, H. T. Yi, S.-W. Cheong, T. Rõõm, Terahertz spectroscopy of spin waves in multiferroic $BiFeO_3$ in High magnetic fields. *Phys. Rev. Lett.* **110**, 257201 (2013).

51. D. Sando, Y. Yang, E. Bousquet, C. Carrétéro, V. Garcia, S. Fusil, D. Dolfi, A. Barthélémy, P. Ghosez, L. Bellaiche, M. Bibes, Large elasto-optic effect and reversible electrochromism in multiferroic $BiFeO_3$. *Nat Commun.* **7**, 10718 (2016).

52. C. Kittel, On the theory of ferromagnetic resonance absorption. *Phys. Rev.* **73**, 155 (1948).
